# Supplementary material for: Integrated stretchable pneumatic strain gauges for electronics-free soft robots
Source: Commun Eng. 2022 Jun 29;1:14. doi: 10.1038/s44172-022-00015-6 (PMC10955973; doi:10.1038/s44172-022-00015-6)
Supplement: Supplementary file 2 — Supplementary Information [file 44172_2022_15_MOESM2_ESM.pdf]

Integrated Stretchable Pneumatic Strain Gauges for Electronics-Free Soft Robots

Anastasia Koivikko, Vilma Lampinen, Mika Pihlajamäki, Kyriacos Yiannacou, Vipul Sharma and Veikko Sariola

Supplementary Materials

Supplementary Tables

Supplementary Table 1. Recently reported stretchable strain sensors and their characteristics. N.R. = Not Reported

Sensor Materials	Fabrication method	Sensor type	Maximum strain tested (%)	Hysteresis (%)	Gauge factor (GF)	Tested frequency (Hz)	Reference
Ionic solution/Liquid metal/Silicone elastomer	Elastomer casting/Channel filling	Resistive	100	N.R.	~3	N.R.	1
AgNW/elastomer composite	Elastomer casting/nanowire coating	Resistive	150	N.R.	~81	N.R.	2
$Ti_3C_2T_x$-AgNW-based ink/polyurethane	Screen-printing	Resistive	83	~20*	200-8700	1	3
Liquid metal/silicone elastomer	Elastomer casting/Channel filling	Resistive	550	N.R.	4.95	N.R.	4
Graphite and carbon black nanoparticles/cotton	Manual coating	Resistive	400	~0**	~100,000	0.2	5
Silver flake ink/thermoplastic polyurethane	Screen-printing	Resistive	2	17	5.8	N.R.	6
Ionigel coped with Fe_3O_4 particles	Synthesis of nanoparticles	Resistive	2000	~13***	20	0.005***	7
Ionic hydrogel/Silver nanofiber	Manual stacking	Capacitive	1000	~0****	165	0.08	8
Expanded intercalated graphite/fabric	Direct writing, screen-printing	Capacitive	250	~0	~0.5-1.2	0.75	9
Parylene/Gold film/VHB elastomer	Manual stacking	Capacitive	140	~0	~3	N.R.	10
**Silicone elastomer**	**Elastomer casting**	**Pneumatic Resistance**	**300**	**~0*******	**~1**	**1**	**This work**

*Estimated from Fig. 5c in[3]
**Until 75%, then hysteresis observed
***Estimated from Fig. S11 in [7]
****100% strain, some hysteresis with 400% strain
*****Measured in 50%

Supplementary Figures

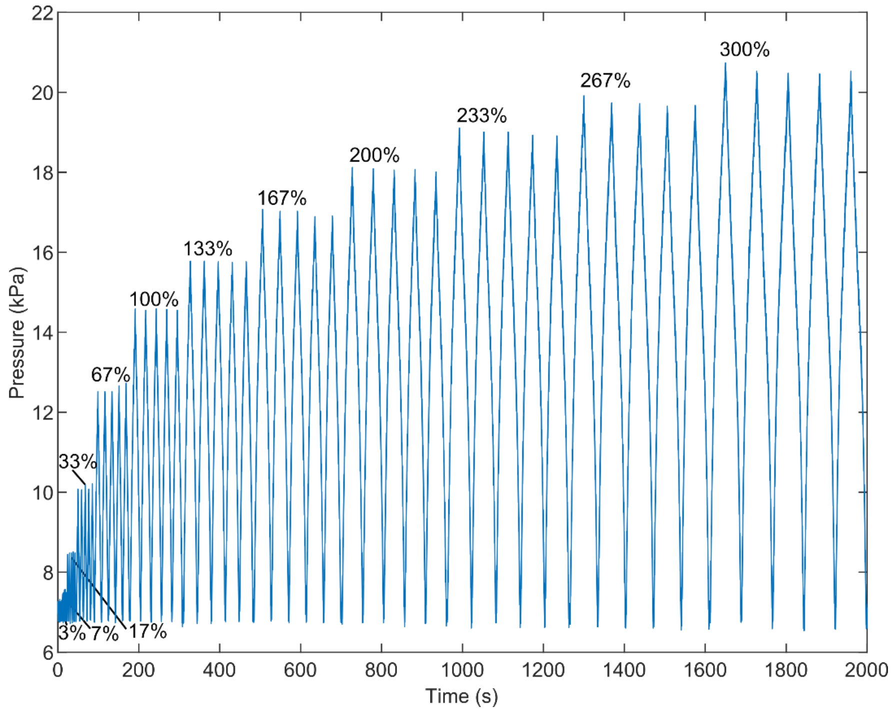

Supplementary Figure 1. Pressure in the pneumatic strain gauge under varying engineering strain.

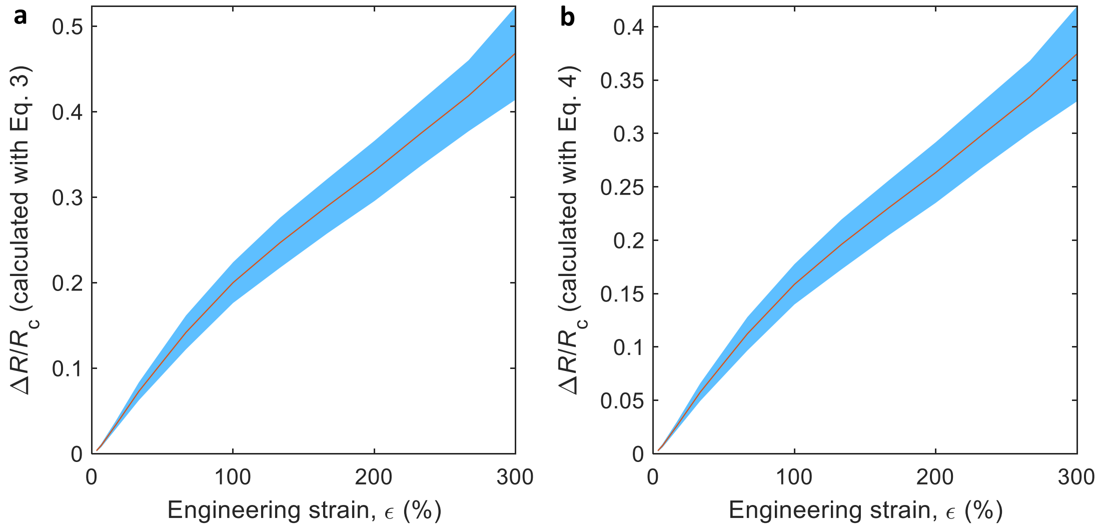

Supplementary Figure 2. Relative change in pneumatic resistance using square pressure equations for compressible fluids (Eq. (3) in **a**) and the small pressure approximation (Eq. (4) in **b**). $\Delta R/R_c$ is shown as a function of engineering strain. The shaded area shows standard deviation.

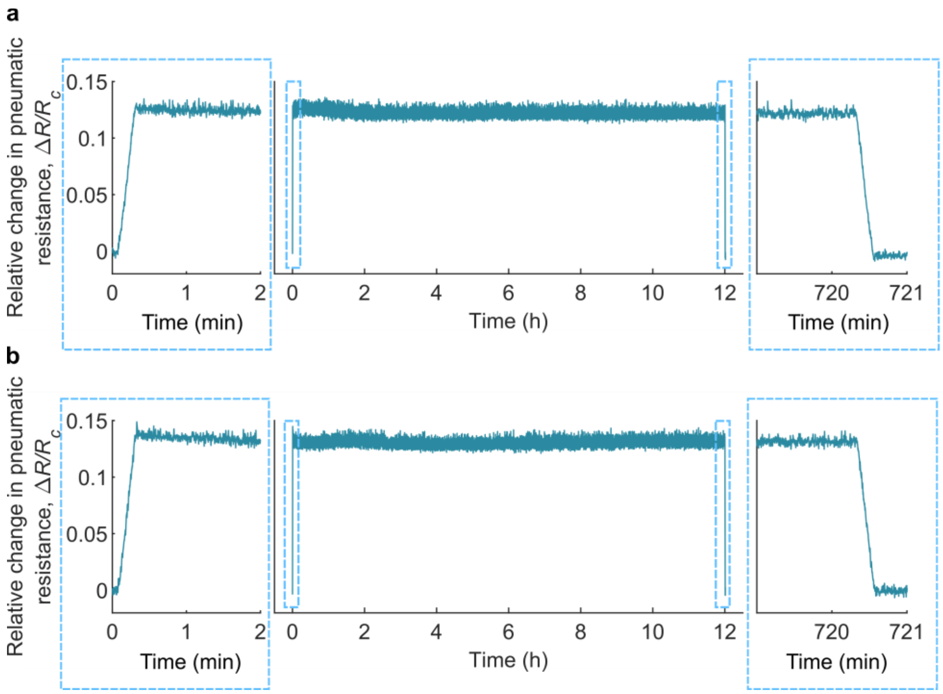

Supplementary Figure 3. Long term stability measurements reported in Fig. 2a repeated for two other samples (**a**&**b**) with the same design.

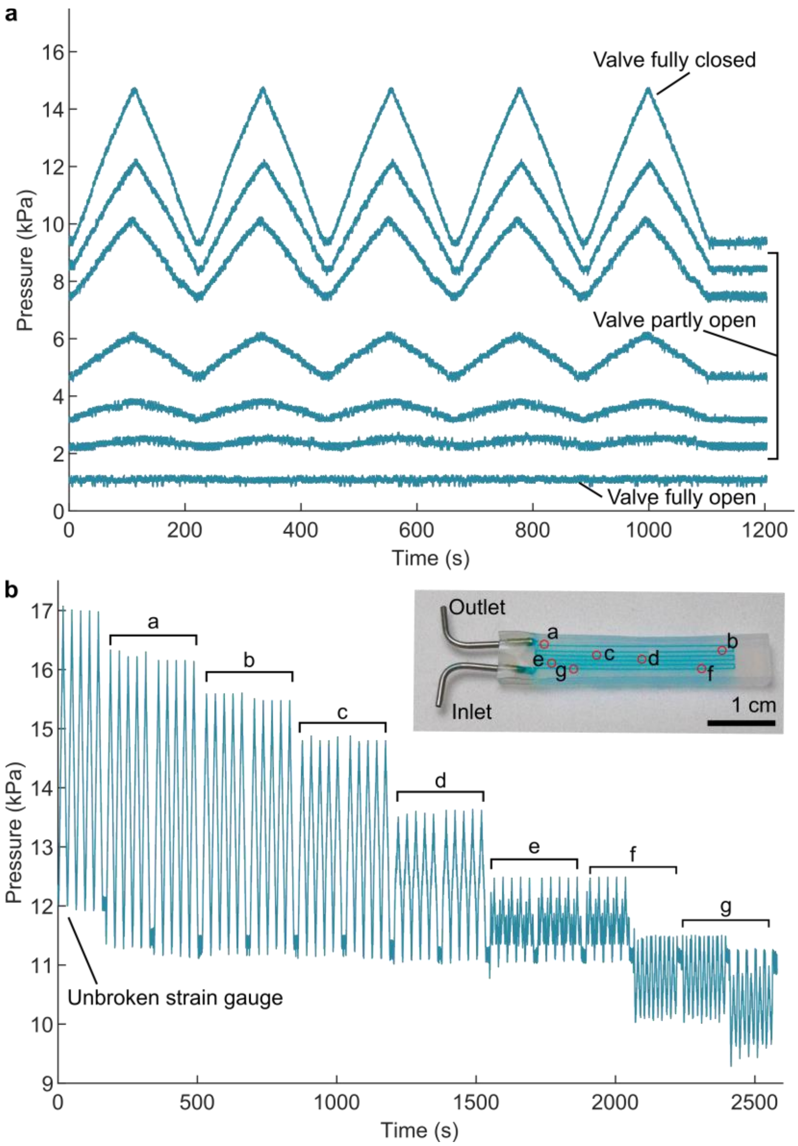

Supplementary Figure 4. Behavior of pneumatic strain gauge in two types of artificial leaks: **a**, pneumatic valve was added in parallel with the strain gauge and it was opened with varying degrees. **b**, The strain gauge was punctured at different positions (inset). At each position, the strain gauge was first punctured with a 0.3 mm needle, and then with a 0.5 mm needle. After each puncture, the strain gauge response was tested for 5 cycles.

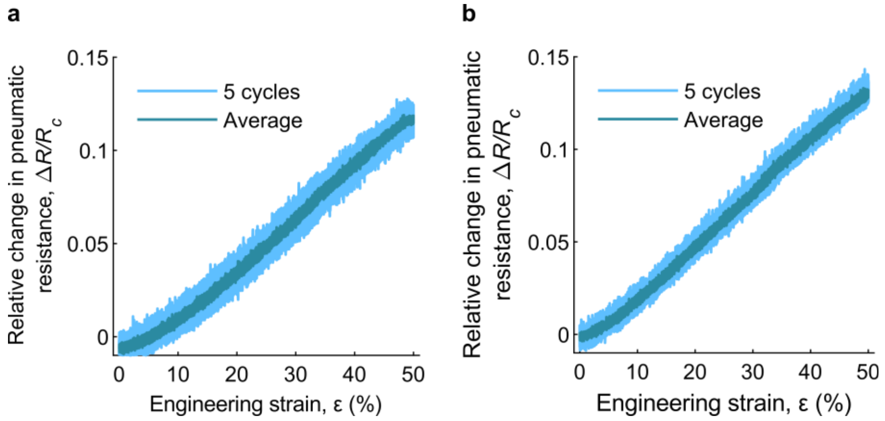

Supplementary Figure 5. Hysteresis measurements reported in Fig. 2c repeated for two other samples **a** and **b** with the same design.

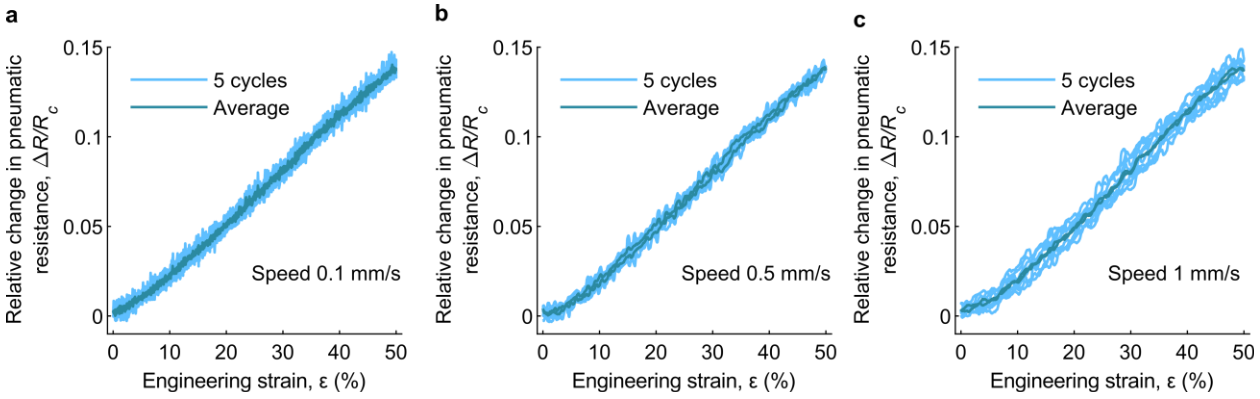

Supplementary Figure 6. Rate-dependent hysteresis of the strain gauge. The gauge was strained with a speed of **a** 0.1 mm s^{-1}, **b** 0.5 mm s^{-1} and **c** 1 mm s^{-1}.

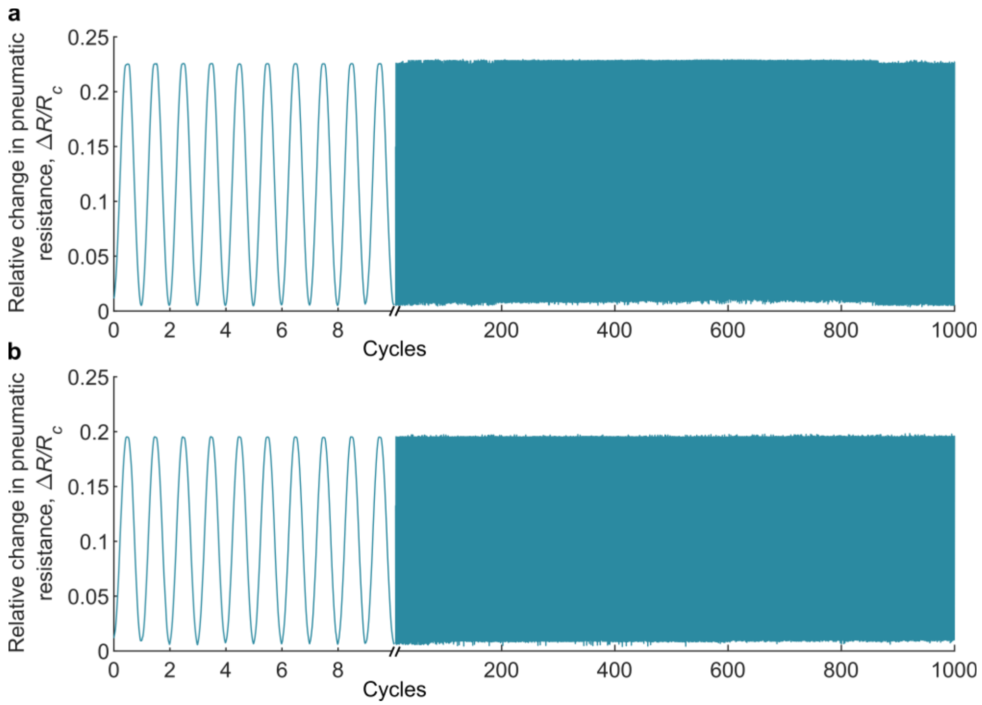

Supplementary Figure 7. Cyclic strain measurements reported in Fig. 2d repeated for two other samples **a** and **b** with the same design.

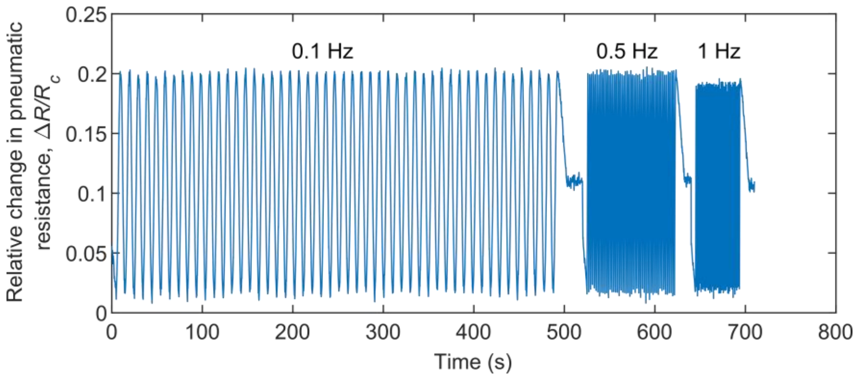

Supplementary Figure 8. Strain gauge under 66.7% cyclic strain with an increasing frequency.

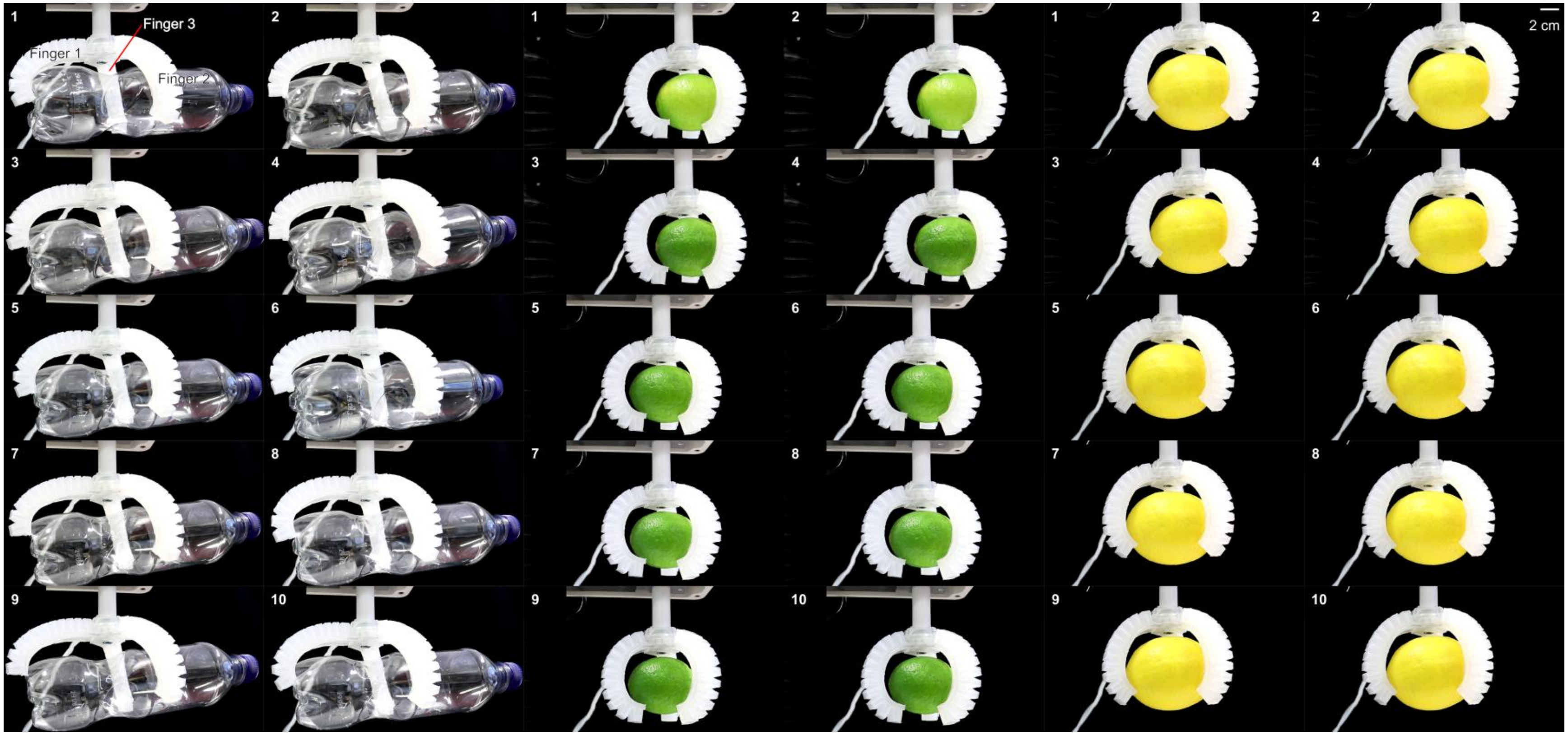

Supplementary Figure 9. Snapshots from each picking experiment (1-10) of the water bottle, the lime, and the lemon in Fig. 4c.

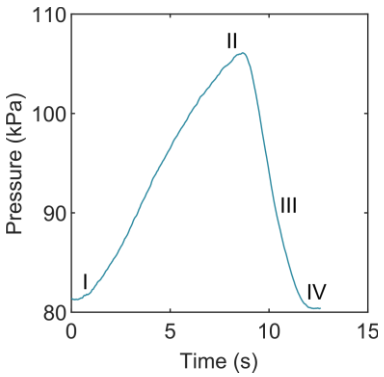

Supplementary Figure 10. Pressure change inside the actuators of the gripper fingers. The pressure changes because the resistance of the tactile sensor (R_4 in Fig. 4a) changes. The roman numerals correspond to the snapshots in Fig. 4d.

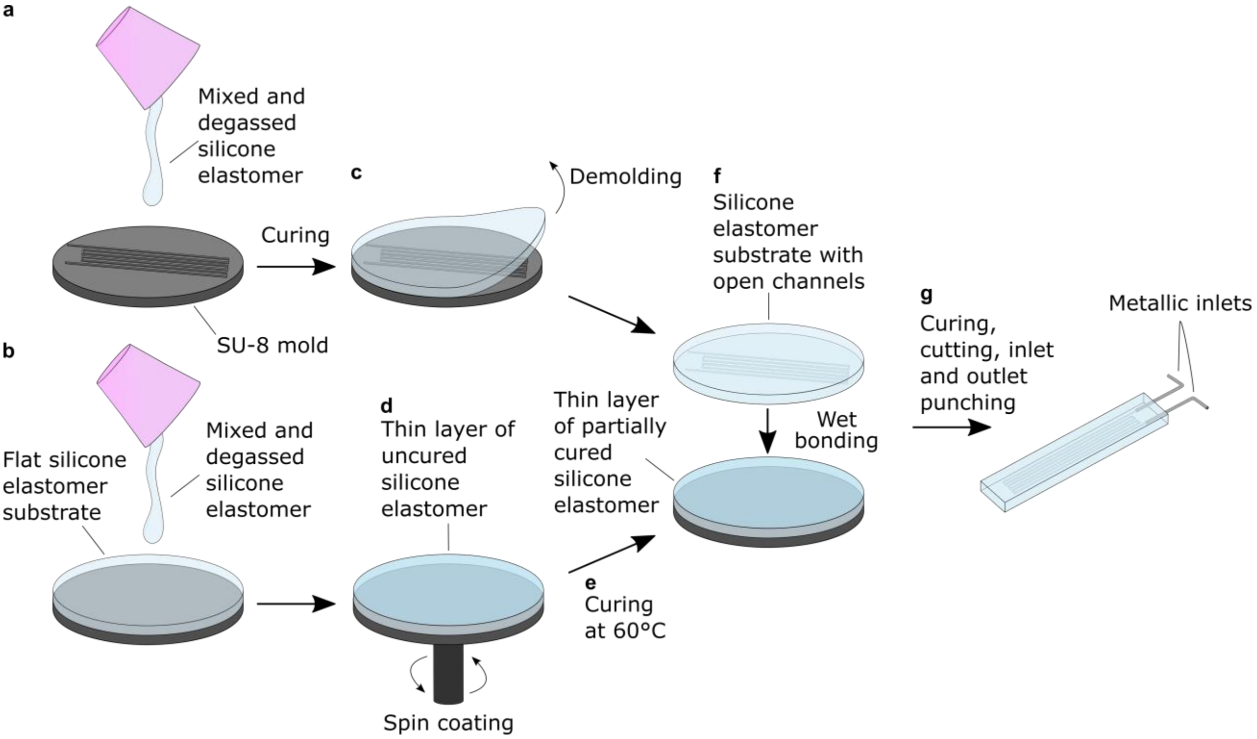

Supplementary Figure 11. Fabrication of the soft pneumatic strain gauges. First, two elastomer sheets are fabricated: one with open channels (**a**) and the other one without any structures (**b**). After curing, the sheet with the channels was demolded (**c**). The flat elastomer sheet was placed in a spin coater and a thin layer of uncured elastomer was coated on top of it (**d**). Then, the flat sheet with uncured elastomer was placed in an oven for 40 s at 60°C (**e**). Next, the elastomer sheet with the channels was placed on top of the uncured elastomer and let cure (**f**). The strain gauges were then cut from the elastomer piece (**g**). Holes for the inlet and the outlet were pierced with a needle and metal inlets were inserted into them.

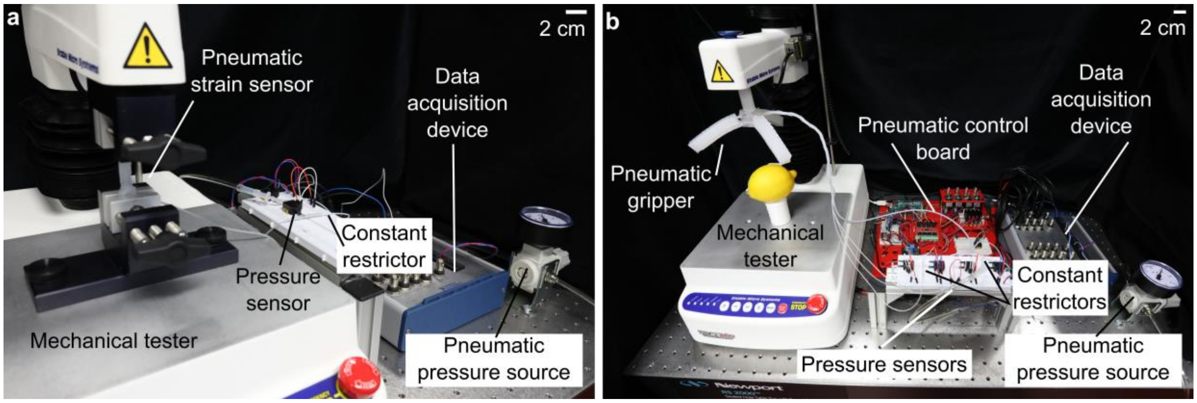

Supplementary Figure 12. Measurement setup **a,** for the strain gauge and **b,** the soft pneumatic gripper.

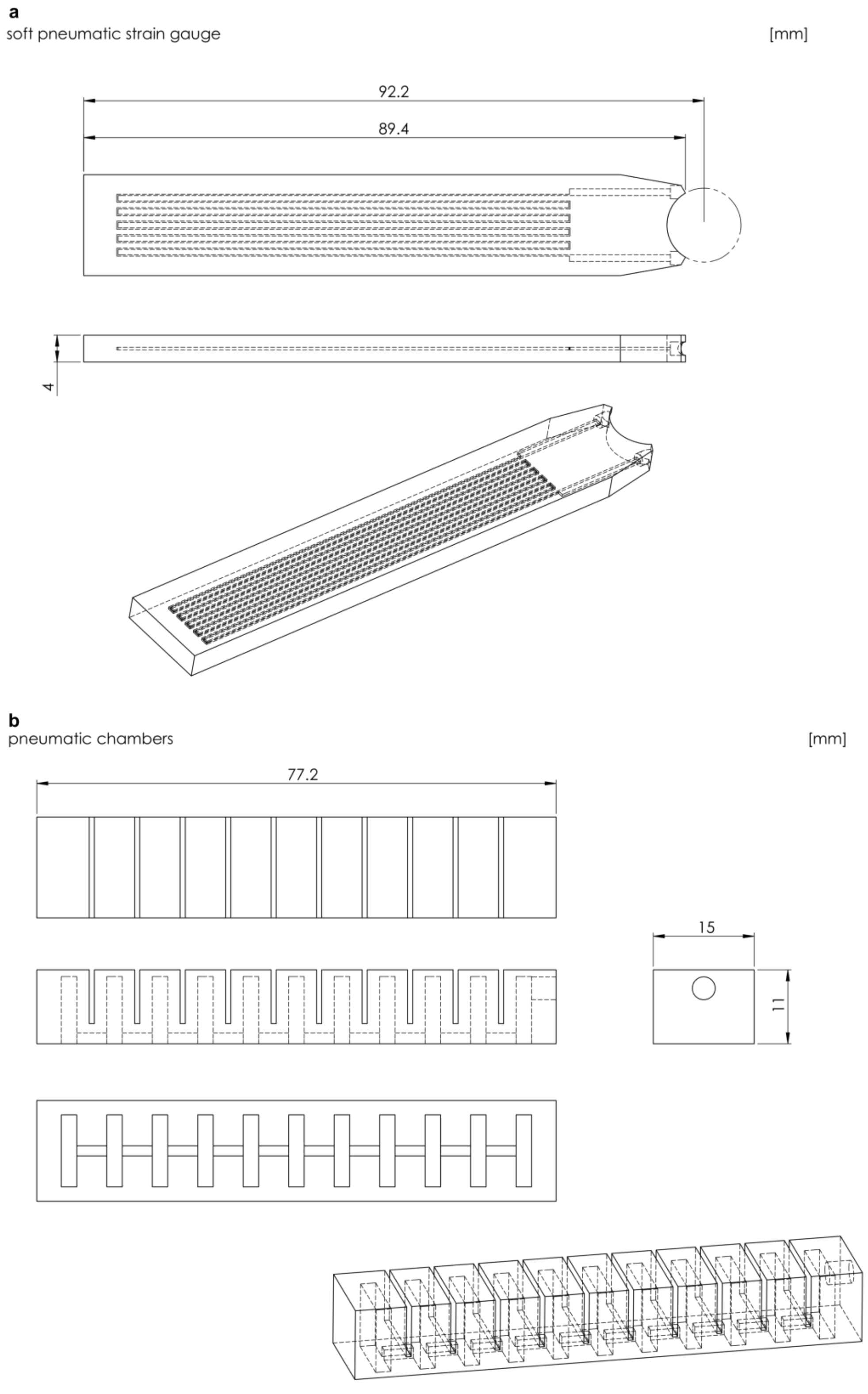

Supplementary Figure 13. Detailed drawings of the soft pneumatic actuator with an integrated pneumatic strain gauge. **a**, the lower part of the actuator with the soft pneumatic strain gauge and **b**, upper part with pneumatic chambers.

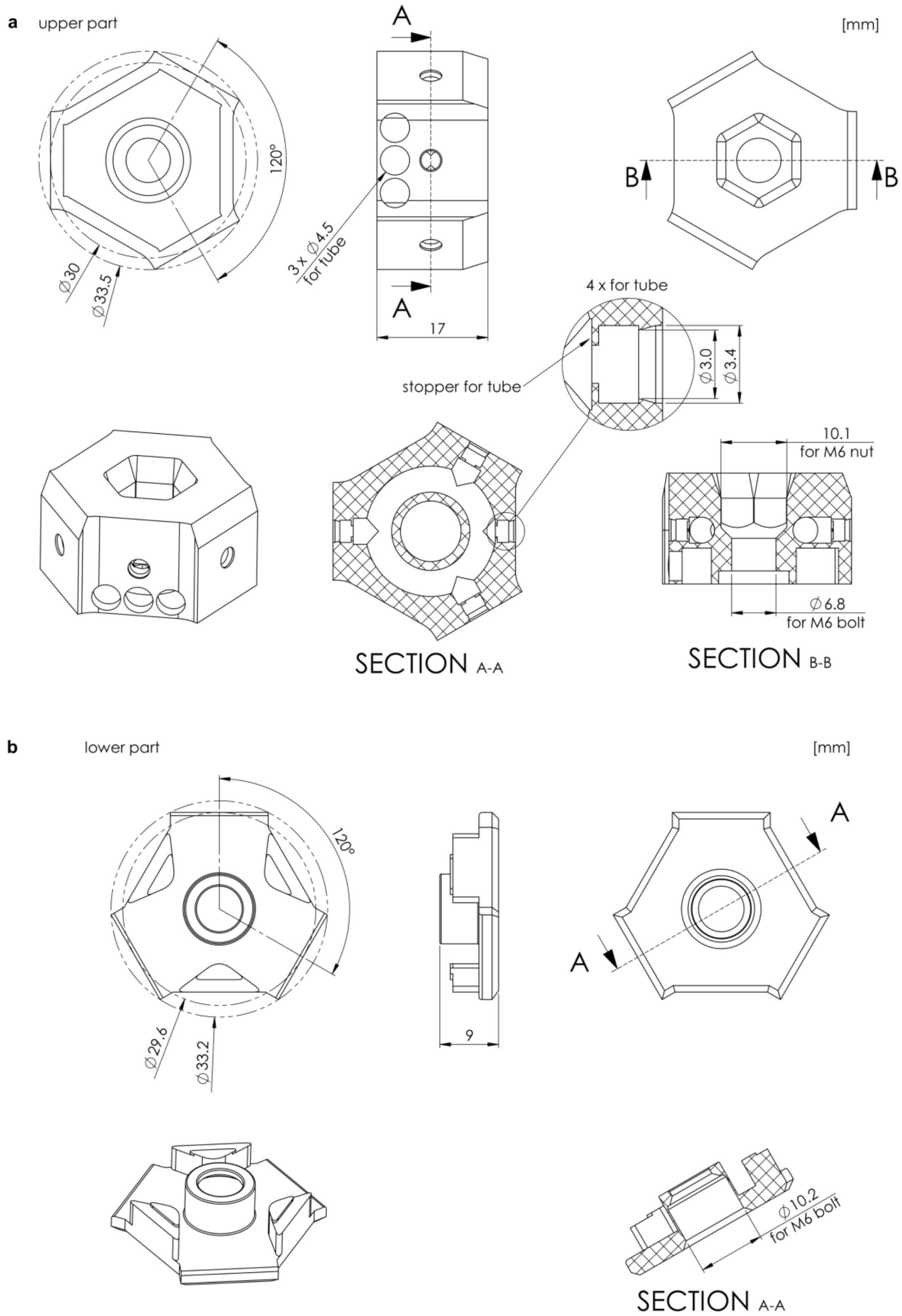

Supplementary Figure 14. Detailed drawings of the 3D printed connecting piece in the soft gripper. **a** is the upper part of the connecting piece and **b** the lower part.

Supplementary References

1. Chossat, J.-B. B., Park, Y.-L. L., Wood, R. J. & Duchaine, V. A Soft Strain Sensor Based on Ionic and Metal Liquids. *IEEE Sens. J.* **13**, 3405–3414 (2013).

2. Kim, K.-H., Jang, N.-S., Ha, S.-H., Cho, J. H. & Kim, J.-M. Highly Sensitive and Stretchable Resistive Strain Sensors Based on Microstructured Metal Nanowire/Elastomer Composite Films. *Small* **14**, 1704232 (2018).

3. Shi, X. *et al.* Bioinspired Ultrasensitive and Stretchable MXene-Based Strain Sensor via Nacre-Mimetic Microscale “Brick-and-Mortar” Architecture. *ACS Nano* **13**, 649–659 (2018).

4. Gao, Q. *et al.* Microchannel structural Design For a Room-temperature Liquid Metal Based super-stretchable sensor. *Sci. Rep.* **9**, (2019).

5. Souri, H. & Bhattacharyya, D. Highly sensitive, stretchable and wearable strain sensors using fragmented conductive cotton fabric. *J. Mater. Chem. C* **6**, 10524–10531 (2018).

6. Koivikko, A., Sadeghian Raei, E., Mosallaei, M., Mäntysalo, M. & Sariola, V. Screen-printed curvature sensors for soft robots. *IEEE Sens. J.* **18**, 223–230 (2018).

7. Mei Zhang, L. *et al.* Self-Healing, Adhesive, and Highly Stretchable Ionogel as a Strain Sensor for Extremely Large Deformation. *Small* **15**, 1804651 (2019).

8. Xu, H. *et al.* An ultra-stretchable, highly sensitive and biocompatible capacitive strain sensor from an ionic nanocomposite for on-skin monitoring. *Nanoscale* **11**, 1570–1578 (2019).

9. White, E. L., Yuen, M. C., Case, J. C. & Kramer, R. K. Low-Cost, Facile, and Scalable Manufacturing of Capacitive Sensors for Soft Systems. *Adv. Mater. Technol.* **2**, 1700072 (2017).

10. Nur, R. *et al.* A Highly Sensitive Capacitive-type Strain Sensor Using Wrinkled Ultrathin Gold Films. *Nano Lett.* **18**, 5610−5617 (2018).
