# Supplementary material for: Integrated stretchable pneumatic strain gauges for electronics-free soft robots
Source: Commun Eng. 2022 Jun 29;1:14. doi: 10.1038/s44172-022-00015-6 (PMC10955973; doi:10.1038/s44172-022-00015-6)
Supplement: Supplementary file 5 — Description of Additional Supplementary Files [file 44172_2022_15_MOESM5_ESM.pdf]

Description of Additional Supplementary Files

File Name: Supplementary Video 1

Description: Soft pneumatic gripper with proprioception. All three gripper fingers have an integrated strain gauge. The shape of the picked object can be detected by measuring the curvature of the fingers.

File Name: Supplementary Video 2

Description: Self-closing soft pneumatic gripper. A strain gauge is placed onto the gripper palm and the palm sensor is connected in paralle l with the pneumatic fingers. When the sensor resistance increases, the pressure inside the fingers increases, causing the fingers to bend. When the external compression is released, the fingers return to their initial shape
